# An Interference-Managed Hybrid Clustering Algorithm to Improve System Throughput

**DOI:** 10.3390/s22041598

**Published:** 2022-02-18

**Authors:** Naureen Farhan, Safdar Rizvi

**Affiliations:** Department of Computer Science, Bahria University, Stadium Rd., Karachi 44000, Pakistan; safdar.bukc@bahria.edu.pk

**Keywords:** hybrid clustering, interference, throughput, three-tier heterogeneous network (HetNet)

## Abstract

In the current smart era of 5G, cellular devices and mobile data have increased exponentially. The conventional network deployment and protocols do not fulfill the ever-increasing demand for mobile data traffic. Therefore, ultra-dense networks have widely been suggested in the recent literature. However, deploying an ultra-dense network (UDN) under macro cells leads to severe interference management challenges. Although various centralized and distributed clustering methods have been used in most research work, the issue of increased interference persists. This paper proposes a joint small cell power control algorithm (SPC) and interference-managed hybrid clustering (IMHC) scheme, to resolve the issue of co-tier and cross-tier interference in the small cell base station cluster tiers. The small cell base stations (SBSs) are categorized based on their respective transmitting power, as high-power SBSs (HSBSs) and low-power SBSs (LSBSs). The simulation results show that by implementing the IMHC algorithm for SBSs in a three-tier heterogeneous network, the system throughput is improved with reduced interference.

## 1. Introduction

In the context of future networks, a high data rate is the key requirement for the construction of smart cities, highlighting the significance of 5G networks and other advanced technologies. By implementing 5G ultra-dense networks, the usage of different indoor and outdoor applications and services will also increase, because applications in health, sports, monitoring and managing data collections, and precision agriculture, among others, require high data rates to work efficiently. A multi-tier heterogeneous network is the vital component required to fulfill the exponentially increasing user requirements. These multi-tier future networks also require an efficient controlling and managing mechanism to be implemented at multiple levels in the network.

In conjunction with the benefits of future networks, a number of challenges also exist, the most significant of which is to achieve an efficient throughput. An ultra-dense 5G network consists of high-powered macrocell base stations (MBSs) employed as a sink for numerous densely deployed small cell base stations (SBSs). The SBSs transmit at different power levels i.e., micro, pico, and femto base stations (BSs), which are the commonly used SBSs in three-tier heterogeneous network architectures. In this paper, we categorize the SBSs as high-power SBS (HSBS) and low-power SBS (LSBS) nodes serving users at multiple tiers.

These SBSs are deployed randomly and ensure the enhanced capacity is achieved at the cost of increased interference in the network. High interference is the result of the dense and random deployment of SBSs. To reduce this interference in the network, extensive work has already been conducted, and efforts are focused on designing various interference mitigating techniques. Among the existing interference management techniques, clustering has proven to be the most promising approach to achieve an improved data rate with reduced interference [1,2]. In principle, co-tier interference occurs between BSs serving on the same tier, whereas cross-tier interference occurs between the BSs located on different tiers [3].

### 1.1. Motivation

Improved throughput can be attained by reducing interference at the user’s end, and extensive research has already been conducted on clustering and other methods to improve network capacity and throughput. Nonetheless, there is a need to undertake research and investigate methods considering the deployment of small cell base stations. If SBSs receive efficient power and signals in a multi-tier heterogeneous network, then the users connected to these SBSs can be served and managed proficiently. Clustering is performed with either a centralized or distributed approach, and rarely with a hybrid scheme. However, there is a requirement for a hybrid clustering technique to significantly reduce interference at SBSs (per tier level) in a multi-tier heterogeneous network to achieve improved throughput and capacity in UDNs.

### 1.2. Prior Work

Clustering has emerged as a proficient means to manage UDNs in an organized manner by forming clusters. Interference management, frequency management, and power optimization can be performed more competently. Extensive work has already been performed in the relevant fields to improve wireless network performance. Furthermore, targeting the selection of an efficient method for radio resource management, the authors in [2] critically analyzed the existing techniques, including clustering, frequency reuse, graph theory, power minimization, and stochastic geometry to attain efficient radio resource management in heterogeneous networks. Through literature analysis, Farhan et al. [1,2] verified that by implementing clustering in a heterogeneous wireless network, efficiency in scalability, fairness, and an adaptive solution with reduced complexity can be achieved. The joint clustering algorithms implemented with the parameter controlling metrics and graph theory in [2,4,5,6,7] are also termed hybrid clustering methods in the literature, in addition to the algorithms proposed as an arrangement of centralized and distributed clustering algorithms in a single, hybrid scheme.

In the research presented in [8], the author proposed a distributed clustering algorithm with mixed-integer NP-Hard complexity. The problem is divided into two sub-problems: first, the decision of resource allocation and, second, offloading computations. The particle swarm optimization algorithm is used to reduce the overall computing overhead of mobile devices.

Similarly, in [9], multilayer user clustering and scheduling were performed in a distributed manner to increase capacity and improve fairness in a dense radio access network. In reference [10], the author proposed a distributed clustering algorithm, FCRA, and resolved the resource contention problem and interference issue after forming disjoint clusters. In comparison, the concept of enhanced interference coordination was proposed in [11] for radio resource management (RRM) in UDNs. The authors used the distributed MIMO technique to integrate the classification of user equipment.

Therefore, through distributed clustering, the CMs can decide based on the information collected from the neighboring CMs and form clusters with elected CHs. The advantages include energy-efficient clusters with adaptive and dynamic environment requirements. However, distributed clustering also involves a few limitations, such as non-uniform CH distribution and clustering overhead [12].

Contrary to this, research on centralized clustering was performed in [13,14] with SBSs using femtocell base stations. The authors performed radio resource management using SBSs in 5G HetNets through spectrum sharing and network slicing. Furthermore, research work on RRM using SBSs or MBSs with centralized clustering is shown in Table 1.

The sink node collects and processes data from the cluster head (CH) node in a centralized clustering approach. Whereas the CH is responsible for transmitting signals and providing coverage to all cluster members(CM) base stations serving within the cluster, CMs give coverage to all UEs of the same cluster. In [15], the author performed hierarchical agglomerative clustering in a wireless network as an energy efficient technique and formed energy efficient clusters with increased network lifetime. The presented scheme was verified with dendrograms giving quantitative results, in addition to simulation findings. Centralized clustering provides the advantages of an optimum number of CH deployment, reduced overhead, uniform CH distribution, and multi-level clustering [12]. However, there are a number of limitations; for example, low scalability, compromised efficiency, and making early decisions about cluster formation [12] are common issues with centralized clustering.

In [16], the author considered the repulsion feature of BS deployment in the SBS tier of HetNet. They first adopted a HetNet model by applying the Poisson point process (PPP) with the repulsion function and then resolved the resource allocation problem with the ‘Hybrid Clustering Game’ (HCG) by improving throughput and capacity in the network. The author generated an interference graph and then mitigated the interference.

Moreover, other techniques also exist that are used to reduce interference in 5G HetNets to achieve a smart living concept. Commonly, non-orthogonal multiple access (NOMA), Internet-of-Things (IoT), and multi-cell environments have strong potential to transform future living ideas. However, these works are more commonly implemented to improve the user association with the relevant base stations in the heterogeneous networks. The work performed in [17] discusses the importance of non-orthogonal multiple access (NOMA) to achieve smart city concepts, intelligent transportation, and smart manufacturing industries. In particular, the authors of [17] proposed a four-layered architecture based on heterogeneous IoT (HetIoT), consisting of sensing, cloud computing, networking, and applications.

Interference management and power allocation were also proposed in [18] to investigate an efficient resource optimization framework with the improved IoT’s spectral efficiency (SE) under the power domain NOMA. Khan et al. proposed a novel solution that utilizes the NOMA protocol to improve SE for the maximum number of IoT devices of individual frequency blocks by controlling and managing the power received among different IoT devices and ensuring successive interference cancellation (SIC) at the receiving ends.

Subsequently, in [19,20], the author implemented NOMA-enabled back-scattered V2X on a vehicular network to achieve improved system data rates, enhanced energy efficiency with NOMA, and supported connectivity of low-powered Internet-of-Vehicle (IoV) for a 6G transportation system. The results were verified through simulations to demonstrate an efficient scheme that outperformed the results gained with a conventional sub-optimal NOMA protocol. In [21], the authors performed a critical review of the NOMA protocol and proposed a solution to achieve better network performance in terms of interference management, bit error rate (BER), capacity, and energy efficiency using the NOMA protocol. It can be deduced from the previous research work that NOMA contributes to wireless networks in terms of efficient access technology, considering the user association with base stations. In [22], energy efficiency is maximized by a joint solution for efficient device association and a power control mechanism based on Karush–Kuhn–Tucker conditions, and the approach was verified with simulations. In [23], power-controlled interference management for an uplink NOMA-enabled multi-cell heterogeneous environment was performed with Nakagami-m faded links.

Contrary to the aforementioned approaches, the author of [24] used intelligent reflecting surfaces for wireless networks to achieve a smart radio environment by controlling the channel propagation. The authors performed the research to achieve better channel performance rather than working on receivers or transmitters to gain high data rates in 6G wireless networks.

In [25], threshold-based clustering is performed. This approach initializes the pre-defined threshold distance values allowed for clusters and the minimum cluster size with SBSs. Then, by including the closest point, it forms the disjoint clusters; with this algorithm, the author improved the cluster performance. In [26], the evaluation of coverage probability is undertaken with threshold SINR values. The study also states that the Wyner model is impractical because it sets SIR to be constant. However, in OFDMA systems, the SIR value varies frequently across the cell. In [26], stochastic geometry is used to study the spatial realizations of a network. By comparison, the node locations are determined using the Poisson point process, and clustering is performed in a distributed manner using the Poisson cluster process, with the Matern point process used to improve the coverage probability in a dense heterogeneous network.

In summary, the above investigation of the various existing techniques indicates the clustering technique is the most popular and effective means to achieve improved performance at the base station within the multi-tier HetNet. Table 1 shows an analysis of the recent research conducted to improve network performance using different clustering techniques. It can be determined from Table 1 that extensive work has been conducted using clustering algorithms in either the centralized or distributed manner, whereas a hybrid clustering algorithm has rarely been implemented.

On an individual basis, neither of the two clustering approaches proficiently fulfills the efficient clustering requirement of ultra-dense heterogeneous networks, and eventually results in the need to design a clustering algorithm that achieves the benefits of both clustering approaches, i.e., centralized and distributed, in a hybrid manner for multi-tier heterogeneous networks.

**Table 1:** Existing work on various clustering approaches.

Ref.	Year	Delay	Throughput	Capacity	Interference	Power	HetNet	Hybrid	Centralized	Distributed	Clustering
[27]	2021				√		√	√			√
[28]	2020		√					√			√
[29]	2021		√		√		√	√			√
[30]	2019			√	√			√			√
[31]	2018						√		√		√
[32]	2018	√	√				√			√	√
[33]	2017			√					√	√	√
[34]	2017						√	√			√
[35]	2017						√			√	√
[36]	2017				√		√				√
[37]	2017							√			√
[38]	2017			√				√			√
[39]	2015				√	√			√	√	√
[26]	2015				√		√			√	√
[40]	2014		√		√	√			√		√
[41]	2014				√				√		√
[42]	2013								√	√	√
[43]	2013					√	√			√	√

#### Existing work on Hybrid Clustering

Hybrid clustering provides improved performance in terms of availability, data rate, reliability, and high-speed data transmissions. It emerged as an excellent approach to reduce co-tier interference in a heterogeneous network environment [36]. It comprises attributes of both of the above-mentioned clustering techniques. In a few research articles, clustering is achieved based on a hybrid approach to optimize load balancing. In [44], hybrid clustering provides an optimal solution for sub-channel power allocation and increased user demand in dense HetNets by offering an interference mitigation scheme. A few existing hybrid clustering algorithms are summarized below:

- H K Mean—The Hierarchical K-Means clustering algorithm is a self-decisive hybrid technique used to obtain an optimum number of clusters.
- EEHMC—The energy-efficient, multi-hop clustering technique is used to prolong the network’s life by adding multiple hops between CH and BS in wireless networks.
- MFABC—The Multi-Objective Fractional Artificial Bee Colony technique is another energy-efficient hybrid clustering technique, and is based on the bee colony algorithm.

Similarly, in [37], the author proposed a hybrid approach to achieve energy efficiency under distributed clustering. Furthermore, in [45], the author presented an H K Mean–hybrid clustering scheme and improved the existing K-Means clustering algorithm to identify high-density clusters. In [46], a discussion is presented on determining the optimal number of clusters under the hybrid self-decisive clustering approach, considering the Hierarchical Agglomerative clustering and K-Means clustering algorithms. Distinct evaluation of an optimum number of clusters by both centralized and distributed clustering is a challenge compared to a hybrid technique. Some of the most broadly identified problems identified with hierarchical clustering algorithms have not shown successful results with the outliers in a cluster. According to [46], K-Means is mainly implemented on extensive datasets but the quality is dependent on the value of ‘k’. Additionally, [47] proposed a new metric to estimate the amount of interference in the network by working under a hybrid clustering approach using graph theory.

Thus, it can be observed that clustering is implemented in various ways to achieve effective radio resource management under centralized, distributed, or hybrid clustering architectures. In [43], the author implemented a hybrid of centralized and distributed clustering techniques to achieve efficient radio resource management on HetNet. Research performed with the hybrid clustering methods is shown in Table 2.

**Figure 1:**
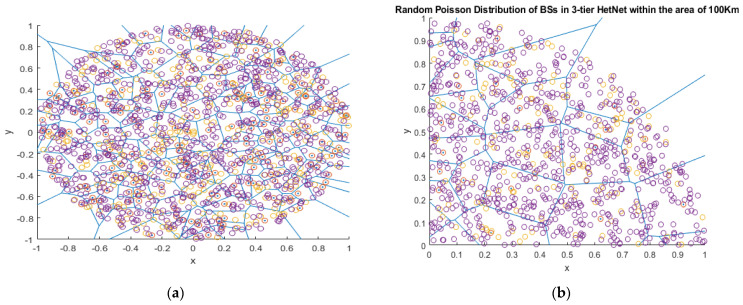
(**a**) A complete view on Voronoi tessellation of a three—tier HetNet with a random Poisson distribution; (**b**) a dissected view of a HetNet with a Poisson random distribution over the area of 100 KM. Red circles with dot represent MBS, yellow circles represent HSBS, and purple circles represent LSBS.

Despite the previous research work undertaken on 5G HetNets to achieve improved performance and efficient radio resource management in clustered architectures, there is still a requirement to investigate clustering methods to improve system throughput and reduce interference in ultra-dense networks. Due to the close deployment of SBSs in ultra-dense HetNets and massive network transmissions, interference at the SBSs increases and requires mitigation to achieve high data rates. This leads to the arrangement of SBSs in such a way that interference can be minimized at both co-tier and cross-tier levels. We propose a hybrid clustering algorithm with a power controlling scheme to reduce interference in SBSs. In Figure 1, the MBS and SBS nodes are deployed randomly under a Poisson distribution on a Voronoi tessellation representing cell areas.

### 1.3. Contribution

This research introduces a hybrid clustering algorithm with an efficient power controlling mechanism. The significant contributions of this paper are as follows:

- A critical analysis of existing cluster-based interference mitigating techniques is performed in our research. Through simulation results, we verified that our proposed hybrid clustering algorithm outperforms the approaches implemented under centralized clustering, distributed clustering, and existing hybrid clustering techniques in terms of interference management and improved system throughput.
- A multi-level clustering technique is proposed to mitigate interference in HetNet. Clustering is applied at multiple layers in a 3-tier heterogeneous network to reduce interference with the proposed IMHC model.
- To manage interference at layer-1 and layer-2, we introduced a power controlling algorithm (SPC), which enables the SBSs to achieve the target SIR threshold value at a minimum transmit power.
- The SIRs achieved with HSBSs (pico BSs) and LSBSs (femto BSs) are compared, and it was verified through simulation results that better signal power is achieved with HSBSs compared to LSBSs. This implies that better throughput is achieved by deploying dense HSBSs in a multi-level heterogeneous network.

### 1.4. Organization

This paper is structured as follows. In Section 2, the system model and problem formulation are explained. In Section 3, the interference-managed hybrid clustering (IMHC) algorithm and small cell power control algorithm (SPC) are explained. In Section 4, the results and simulation methodology, and the simulation parameters, are presented. Finally, the last section presents the conclusion and future dimensions of this research.

## 2. System Model and Problem Formulation

**Figure 2:**
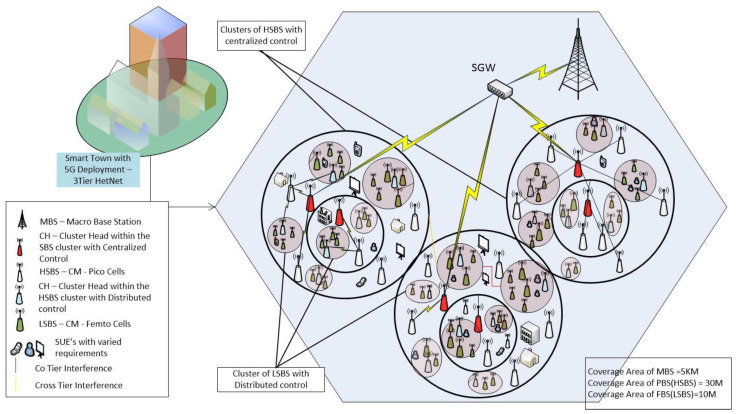
IMHC HetNet Model.

We designed a three-tier heterogeneous cellular network consisting of a macro cell and small cell base stations, as shown in Figure 2. In the IMHC model, clustering is performed at two levels. Moreover, an interference mitigation algorithm SPC is implemented to achieve reduced interference in a three-tier clustered heterogeneous network. The IMHC network is comprised of MBSs at tier-1 and with a random number of SBSs deployed at tier-2 and tier-3 consisting of pico base stations (HSBSs) and femto base stations (LSBSs), respectively. The MBSs are overlaid by randomly distributed SBSs, whereas the SBSs of each tier differ in terms of transmit power, node densities, and link reliabilities. Throughout this paper, we use the terms HSBS and LSBS to categorize high-powered SBSs and low-powered SBSs, respectively.

### Node Deployment and Performance Metrics

The selection of the serving BS amongst the candidate BSs depends on the clustering method applied at the relevant tier. The IMHC scheme is comprised of the following considerations.

**Figure 3:**
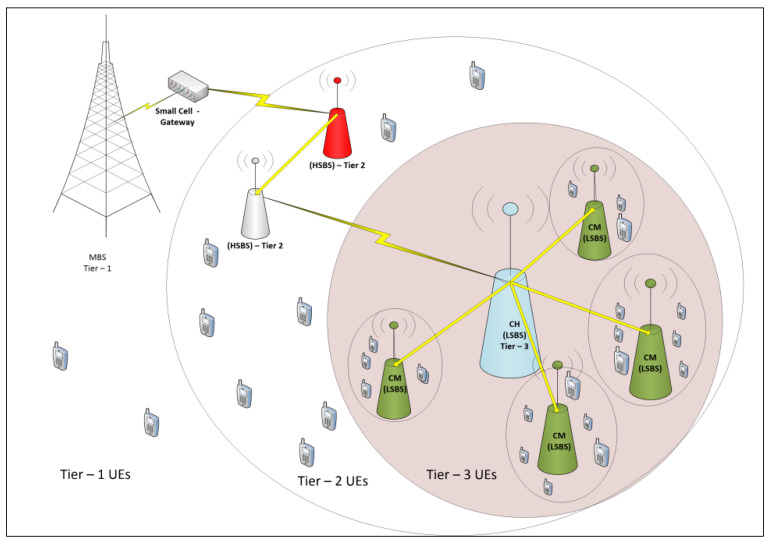
Power dissemination within the 3-tier HetNet.

- Initially consider a network with one MBS deployed per cellular region with the area ‘*A*’; the MBS acts as a sink connected to a small cell gateway (SGW) at tier-1. Assume that the SGW provides the ‘*k*’ number of connections to HSBSs; if ‘*k*’ is the total number of links provided by SGW, then ‘*ka*’ will be the number of active links provided by SGW at tier-2. The SGW act as a central controller to the ‘*k*’ number of nodes of HSBS at tier-2, and CH_H_ to the CM_H_ in the respective clusters, as shown in Figure 3.
- The CM_H_ will act as a sink to the LSBSs. The LSBSs will form distributed clusters with CH_L_ elected by the CM_L_ within the one-hop distance from CH_L_. The HSBSs CHH and CHL radius is denoted by *R_H_* and *R_L_*, respectively. However, the LSBSs coexist within HSBSs with a coverage area less than the coverage area occupied by HSBSs; the topology is shown in Figure 2 and Figure 3.
- Agglomerative clusters of high-power small cell base stations (HSBSs) are formed at tier-2 of the IMHC model with a single linkage. Thus, CH_H_ will be the cluster head and CM_H_ will be the cluster members of tier-2.
- Each HSBS clustered BS (CM_H_) acts as a sink to the low-power small cell base stations (LSBSs). However, at tier-3, the LSBSs are clustered in a distributed manner using the Poisson cluster process. CH_L_ will be elected as cluster head at tier-3 of HetNet based on the highest interference degree by LSBSs as CM_L_ within the one-hop distance of CH_L_.
- Results are generated using MATLAB simulations. Moreover, the results of the IMHC scheme are compared with the results of existing clustering approaches.

The base station locations are deployed under a Poisson distribution with MBS density *λ_m_*, HSBS density *λ_p_*, and LSBS density *λ_f_*. All links are assumed to experience a standard power-law path loss with *α* >= *2*, considering Rayleigh fading (3GPP). If ‘Փ’ represents the Euclidean space, then the received power at a typical receiver from the *ith* tier BS located at a Euclidean distance *|X_i_|* [51] will be:

(1) $$Փ=\sum_{i}\delta x_{i}$$

*P_r_* = *P_i_ h_i_* |*x_i_*|^−*α*^ (2)

where ‘*α*’ is the path loss exponent, ‘*h_i_*’ is the channel gain, and ‘*P_i_*’ is transmit power and is assumed to be constant for the BSs at tier—*i* (*i Є* {*s*,*m*}). The indexes ‘*m*’ and ‘*s*’ denote macro cell tier and small cell tiers, respectively. The candidate serving BS location is:

X*_i_ = *arg max_zi_ _Є_ φ_i_ P_i_*|*x_i_*|^−*α*^ (3)

where X*_i_ is the location of the nearest BS of the *i*th tier (i.e., *φ_i_*) to the typical user. The general SINR expression for MBSs in a heterogeneous network is given as:

(4) $$SINR=P_{r,M}/\sum_{k=1}^{M}I_{M}+\sum_{k=1}^{S}I_{S}+N_{O}$$

where *P_r_*_,*m*_ is the received power at MBS, *I_M_* is the total interference caused by neighboring macro cells’ base stations, *I_S_* is the total interference caused by nearby small cell base stations, and *N_O_* is the AWGN noise factor. In this paper, as we focus on interference, we assume a network where the noise factor is not considered because similar considerations have been made in many research articles by H.S. Dhillon and others [12,37,38]. To define SIR, consider a wireless network of transmitters with positions *x_1_*,*…*,*x_n_* in a region of space. At location ‘*x_i_*’, ‘*P_i_(x)*’ denotes the power of the received signal at location ‘*x*’ from transmitter ‘*x_i_*’.

(5) $$SIR(x,x_{i})=P_{i}(x)/\sum_{j=1}^{n}P_{j}x-P_{i}x$$

According to Equation (4), by increasing the number of base stations/transmitters at location ‘*x*’, the *SIR* value will be decreased, whereas, with a higher number of base stations available for the receiver to be connected, there will be an increased probability of connecting to a base station with a larger ‘*P_i_(x)*’. Thus, the general expression of *SIR* for HetNet will be:

(6) $$SIR=P_{r,M}/\sum_{k=1}^{M}I_{M}+\sum_{k=1}^{S}I_{S}$$

However, the *SIR* expression at a typical SBS ‘*i*’ used in this paper for calculating system *SIR* in a three-tier network is:

(7) $$SIR_{i}=P_{r,i}+h_{i}+|X_{i}|/\sum_{k=1}^{HSBS}I_{H}+\sum_{k=1}^{LSBS}I_{L}+\sum_{k=1}^{z}I_{z}$$

where *P_r_*_,*i*_, *h_i_*_,_ and *|X_i_|* are the transmitting power, channel gain, and position of SBS, respectively, in the intended tier. *I_H_*, *I_L_* represent the total cross-tier interference power from the clustered HSBS and LSBS, respectively, and *I_z_* denotes the aggregated co-tier interference within the same cluster of the SBS ‘*i*’.

It is required to compute throughput coverage probability, usually denoted by ‘*P_c_*’. *P_c_* is formally defined as the probability of SIR that a node experiences by the neighboring nodes is higher than the desired threshold value. It can be given as:

*P_c_* = *Ρ* (*SIR* > *ꞵ*) (8)

where *ꞵ* represents the target *SIR* threshold. Then, throughput can be found by using the following equation. In Equation (7), *λ* represents the total density of simultaneous active SBSs in the per unit area:

*T* = *λ* log 2 (1 + *ꞵ*) *P_c_* (9)

Moreover, signal propagation channel losses and shadowing are common phenomena. Path loss models used primarily on research for wireless communication are Rayleigh fading and Rician fading for non-line-of-sight and line of sight propagation. Path loss occurs due to the dissipation of energy, and depends on the distance between the receiver and transmitter, whereas the shadowing effect occurs due to obstacles and is caused by absorption and reflection of energy. Given Rayleigh fading, the mathematical propagation model consists of the random and deterministic components; the general form can be given as:

*P_i_*(*x*) = *F_i_*/*l_r_* (|*X_i_*|) (10)

where ‘*F_i_*’ is a non-negative random variable and ‘*l_r_*’ is a non-negative path loss function.

*l_r_* = (*kr*)^−*Γ*^   Ɐ (*Γ* > 0) and (*k* > 0) (11)

The function ‘*l_r_*’ is assumed to decrease with the increase in ‘*r*’, as the path loss increases with the increase in distance ‘*r*’. ‘*Γ*’ and ‘*k*’ in Equation (9) are model constants, fitted to real-world data. Channel fading can be categorized as large-scale fading and medium-scale fading with free space losses and shadowing, respectively.

*L_p_* = 20 log 10 (*πλ*/4) + 20 log 10 (*d*) (12)

Typical distributions for fading variables include the exponential and gamma distributions, whereas the log-normal distribution is usually used for shadowing. Maximum fading and shadowing variables are assumed to be independent and identically distributed (i.i.d), whereas rarely random fields are used to include a degree of statistical dependence between variables.

## 3. Interference-Managed Hybrid Clustering (IMHC) Scheme

Consider a wireless network with macro, pico, and femto BSs located randomly in an open access model over area ‘*A*’, with distribution *λ_m_*, *λ_p_*, *λ_f_* densities. All nodes broadcast their power level ‘*P_a_*’ (*P_a_ Є P*) to SGW, whereas SGW forms a list and compares the received *P_a_* value with the threshold power value ‘*P_t_*’. This threshold value ‘*P_t_*’ helps the SGW decide whether the intended SBS will be considered a HSBS or LSBS. The nodes assigned as HSBS will receive the value ‘*k*’ from SGW to form CH_H_. Thus, clusters of HSBS with centralized control will be formed based on their respective Euclidean distances, agglomerative clustering will be performed, and a proximity matrix ‘*W*’ will be formed.

IMHC—Pseudo Code

Small Cell Base Stations (SBSs)

 

*Tier-2*

*Centralized Cluster Formation*

*Randomly Deployed in Euclidean space ‘Փ’*

*1:*  *Let, A = All active nodes, SBS = H ∪ L*
*2:*  *H = All high-power small cell base stations (HSBS)*
*3:*  *L = All low-power small cell base stations (LSBS)*
*4:*  *X = {The position co-ordinates x_v_ of nodes (SBSs) v, x_v_ |v Є H}*
*5:*  *Node v sends its position x_v_ and interference level I_v_ to the Gateway.*
*6:*  *The Gateway performs the following function.*
  *a.*  *Set the weight proximity matrix W based on the Euclidean distance, where W = {w_1_, w_2_, …, w_n_} by random numbers.*
  *b.*  *Sets the target SIR value.*
  *c.*  *Find the nearest w_k_ Є W to x_v_*
*7:*  *For each K Є {1, 2, …, k}, kth CH is assigned to the closest node v Є H. Let, C = {v Є H is CH} be the set of CHs, then, c (k) Є C denotes the Kth cluster.*
*8:*  *All nodes, i.e., v are allocated to Kth cluster, in the intended CH will be the closest CH to v.*
*9:*  *SGW broadcasts the selected CH and cluster assigned CMs. This will also reduce the overhead interference in tier-2.*
*10:*  *All CMs will compute and send their received total interference (I_t_) value to the CH.*
*11:*  *The decision is taken by the CH, such that, **P_i_** (I_t_ <= τ) threshold interference value.*
*12:*  *The interference threshold value is introduced at each CH as the success probability, to mitigate interference.*
*13:*  *Therefore, the total interference will be achieved as: I_t_ = I_M_+I_H_+I_L_*

*Tier-3*

*Distributed Cluster Formation*

*Randomly Deployed*

*1:*  *Each Femto access point FAP (LSBS) begins by listing its respective one-hop neighbor list, comprising of the identity of its respective interfering LSBSs by the sensing environment.*
*2:*  *Every LSBS calculates the number of interfering LSBSs; this parameter can be called the ‘interfering degree’ of each of its one-hop low-powered femto base stations.*
*3:*  *Based on this, CH will be elected and later notified to its respective CMs.*
*4:*  *The LSBS with highest interference degree will be CH, and other one-hop neighbors will be the CMs.*
*5:*  *In the case of a stand-off situation, in which all LSBSs have an equal interference degree, a random tie break will be used, and in the other similar cases, when no node is elected as CH. All neighbors will be associated as CMs to other clusters.*

Due to the agglomerative clustering of HSBSs, there will be interference between the HSBSs. Both co-tier and cross-tier interference will occur among the base stations if HSBSs experience increased interference, which will eventually weaken the transmit power of SBSs to users. Due to a lower transmit power of SBSs to the user, the overall system throughput will ultimately decline.

Therefore, we proposed a small cell power control algorithm (SPC) that enables the SBSs to achieve the target SIR at a minimum aggregate transmit power, assuming that the target SIRs are feasible. However, in the case of the proposed tier-2 agglomerative clustering method, when achieving the target SIR is not achievable by HSBSs, the selected HSBSs will be thinned and considered LSBSs, and will form clusters with LSBSs. Conversely, HSBSs with a target SIR or higher will continue to serve within the same tier, i.e., tier-2 of the HetNet model.

Small Cell Power Control Algorithm for clustered SBSs (SPC):

*Step 1:*  *The target SIR is achieved based on threshold interference value.*
*Step 2:*  *CMs of the intended tier will achieve the target SIR under the assumption that the target SIR is feasible within the achievable service range.*
*Step 3:*  *CMs that achieve the target SIR continue to serve within the same tier.*
*Step 4:*  *CMs that failed to achieve the target SIR will be thinned from the current tier cluster to the cluster of low-powered tier.*
*Step 5:*  *Thus, the outage probability of SBSs with high transmission power will be reduced and improved system throughput will be achieved with clustered SBSs.*

**Figure 4:**
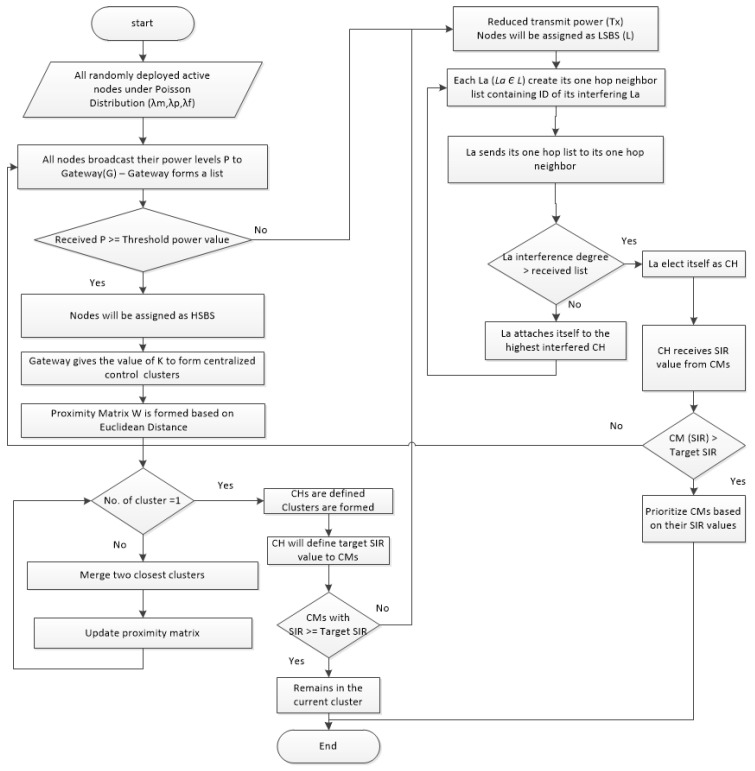
Process flow of the IMHC hybrid clustering scheme.

LSBSs with low transmit power form clusters with distributed control, so that LSBSs will be organized efficiently. This will also reduce interference among the base stations by grouping LSBSs in clusters. However, within the distributed clusters, co-tier interference will arise, but will be managed by implementing the SPC algorithm as shown in the flow diagram, i.e., Figure 4.

Therefore, by clustering the HSBSs in a centralized manner with the SPC algorithm, the co-tier and cross-tier interference is reduced. Furthermore, at the succeeding layer, by clustering the LSBSs once more with the SPC algorithm, the co-tier and cross-tier interference is also reduced. Thus, it will eventually minimize the overall system interference in an ultra-dense heterogeneous network laid on three tiers or more. The pseudo-code for clustering with distributed control is explained below.

## 4. Simulation Methodology and Results

To evaluate the IMHC scheme of the hybrid clustering interference controlled algorithm, MATLAB was used to perform simulations. The overall simulation setup is summarized in Table 3; the values were selected based on 3GPP standards and simulation parameters used in [12,16,52]. All active SBS nodes were deployed randomly using a Poisson random distribution, within a macro cell area.

**Figure 5:**
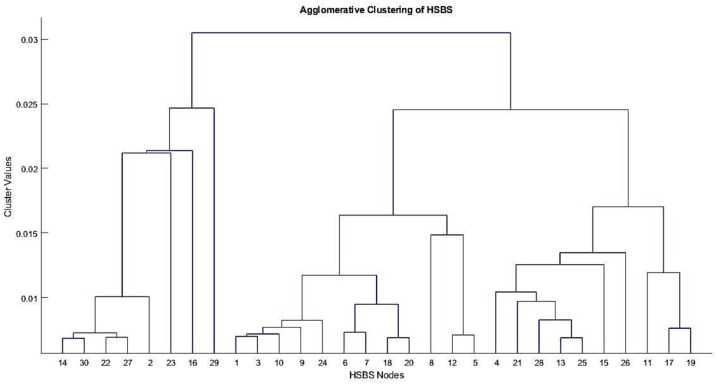
A dendrogram of the HSBS centrally controlled clusters.

**Figure 6:**
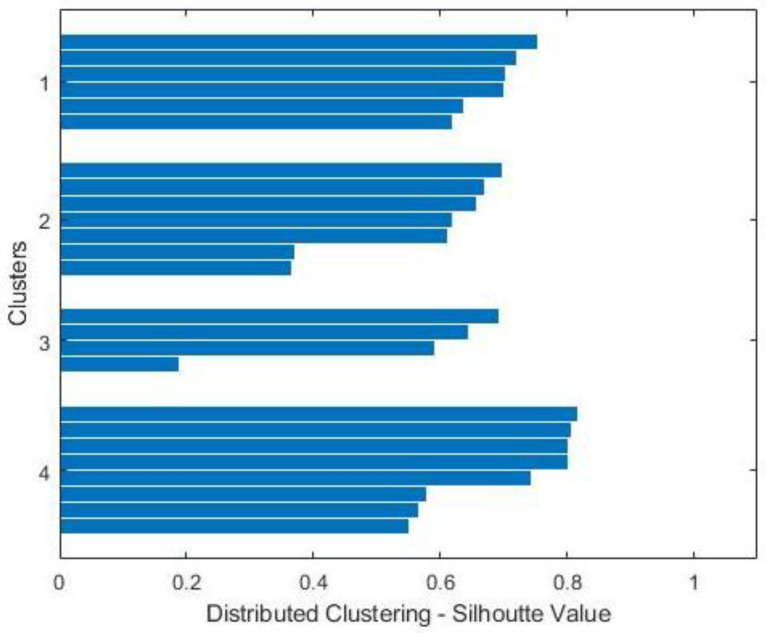
Silhouette representation of distributed clustering of LSBSs.

Figure 5 shows a single linkage dendrogram of agglomerative clusters formed of HSBS nodes. Agglomerative clustering is performed on single and average linkage, but the results with single linkage are found to be better than the average linkage clusters. After forming centralized clusters with a single linkage of random HSBS nodes, we performed the proposed distributed clustering on the acquired values from centrally controlled clusters. Then, we generated the silhouette graphical representation of clusters/partitions to ensure the tightness within the clusters and separation between the clusters. A silhouette representation is a popular machine learning display technique to show mean fairness in the formed clusters [53]. Figure 6 shows the silhouette graph generated for tier-3 LSBSs with the number of clusters, n = 4. It can be observed in the Figure 6 graph that the silhouette value reaches 0.8 and that means fair distributed clustering was performed on the acquired values.

Moreover, the separation between the clusters shows that the probability of interference among LSBS clusters is reduced. It can also be observed in Figure 6 that the clusters are formed with varying densities.

**Figure 7:**
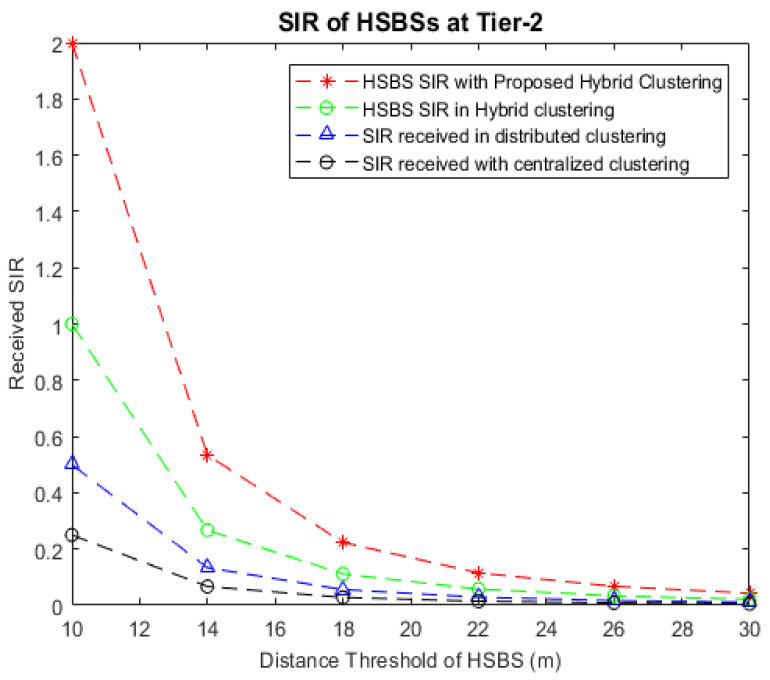
Comparison of received SIR vs. distance in meters at LSBSs with centralized clustering, distributed clustering, existing hybrid clustering, and the IMHC hybrid clustering scheme.

Figure 7 shows simulation results for the received SIR at HSBSs with centralized clustering, distributed clustering, the existing hybrid clustering method, and the IMHC hybrid clustering method. The IMHC hybrid scheme gives better SIR values than other clustering methods for HSBSs that can provide service coverage up to the distance of 30 m. In Figure 7, the received SIR vs. the HSBS service range in meters is given; it can be observed that the IMHC scheme improves the SIR values by 42%, 38%, and 25% compared to centralized clustering, distributed clustering, and the existing hybrid clustering approach, respectively. With the increase in distance, the SIR value decreases for nearly all clustering methods and eventually the service efficiency declines. This suggests that dense clusters with shorter radii should be formed to improve the respective clusters’ data rates.

**Figure 8:**
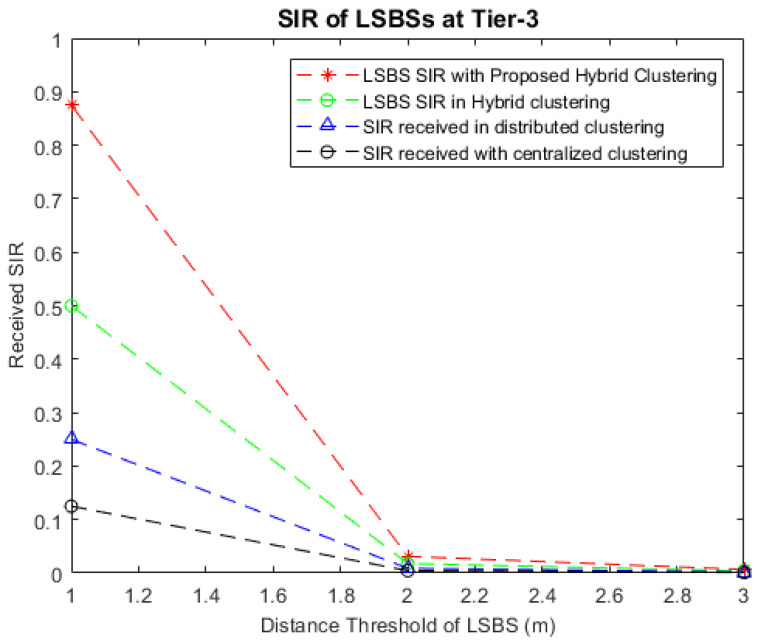
Comparison of received SIR vs. distance in meters at LSBSs with centralized clustering, distributed clustering, existing hybrid clustering, and the IMHC hybrid clustering scheme.

The simulation results in Figure 8 show that better SIR values are achieved with the IMHC hybrid clustering algorithm with LSBSs. It can be observed that, with the shorter service ranges, the IMHC scheme improves the SIR values of LSBSs by nearly 40%, 10%, and 6% compared to centralized clustering, distributed clustering, and existing hybrid clustering, respectively. Thus, reduced interference is achieved in both levels of clustering, i.e., at tier-2 and tier-3, with pico BSs and femto BSs, respectively. Again, it can be observed that the denser the LSBSs clusters deployed, the better the received SIR power.

**Figure 9:**
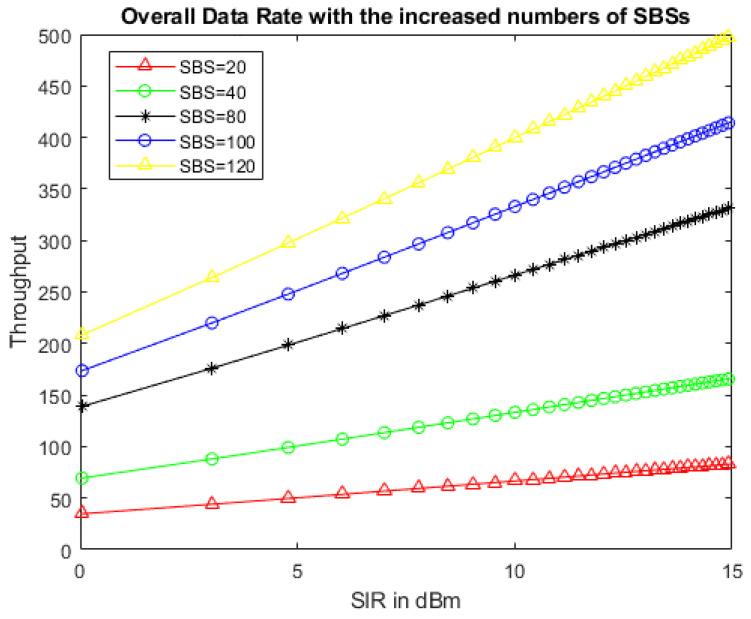
System throughput achieved with the IMHC scheme by increasing the number of SBSs in HetNet.

Figure 9 shows the overall system throughput achieved after implementing the IMHC hybrid clustering scheme of SBSs, i.e., by increasing the number of SBSs, the achieved system throughput was improved significantly.

**Figure 10:**
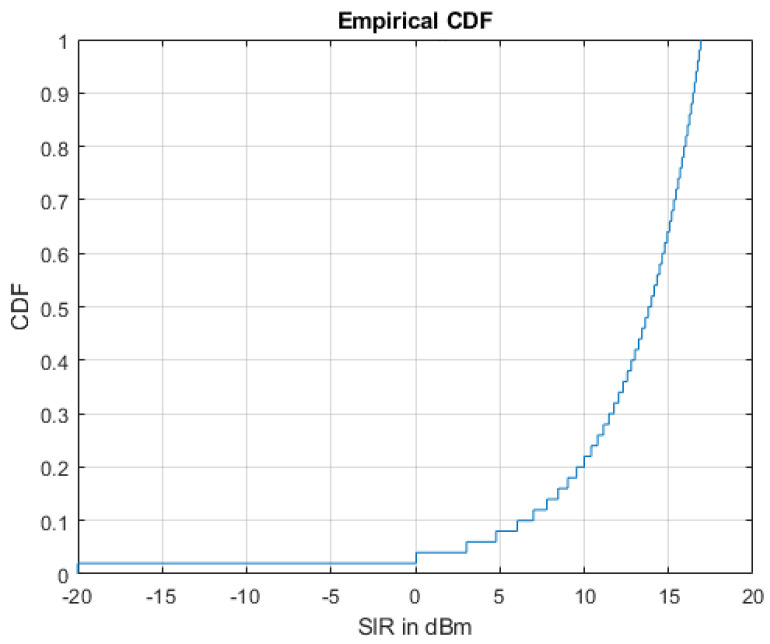
CDF of small cell BSs SIR with the IMHC hybrid clustering algorithm.

**Figure 11:**
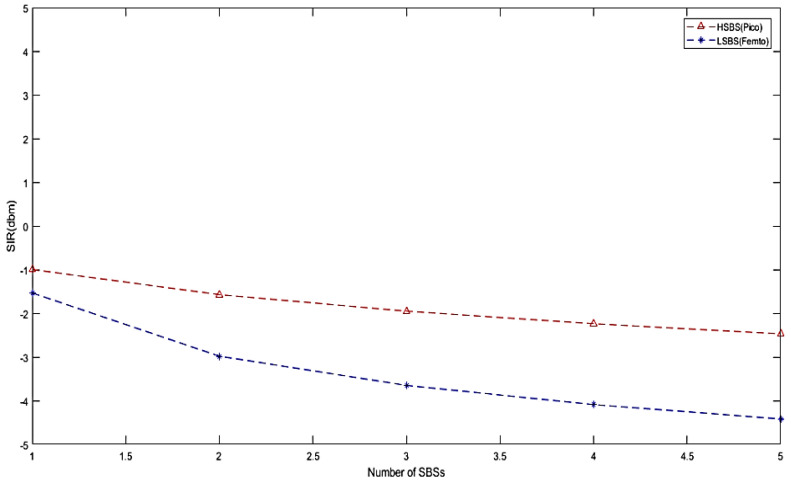
SIR comparison of HSBS and LSBS with the IMHC scheme.

Finally, to validate the obtained results, we generated the CDF of SIRs achieved for the SBSs that performed better in the IMHC hybrid clustering environment, as shown in Figure 10. Moreover, it can be analyzed from Figure 11 that the performance of the IMHC scheme was better with HSBSs than LSBSs. This implies that the IMHC scheme yields a lower outage ratio of HSBSs than LSBSs.

## 5. Conclusions

It can be concluded from the proposed research that, in a heterogeneous network environment, the small cell base stations are deployed closely and randomly under dense conditions in a given area. Therefore, by categorizing the SBSs based on the received power and deploying them in a multi-tier architecture, the interference is reduced in the SBS tiers. In a multi-tier network with BSs having various power values, it is evident that BSs with high-power values will cause more interference than the BSs with low-power values. Therefore, by categorizing the SBSs as high- and low-power SBSs using efficient clustering methods, and implementing the SPC algorithm with the IMHC scheme, interference is managed more effectively and improved network throughput is achieved. In the future, NOMA or other multi-access-based user association schemes under the IMHC scheme can be implemented to achieve improved coverage and capacity.

## Figures and Tables

**Table 2:** Existing hybrid clustering techniques.

Year	Paper	Parameter	Hybrid Clustering Technique
2021	[48]	BER against the achieved SNR values.	Successive interference cancellation technique. NOMA and CDMA. Worked on spread spectrum
2018	[16]	Fairness, spectral efficiency, and improved throughput.	Mitigated interference by implementing Hybrid clustering game algorithm based on Matern Hard core process (MHP). Spectrum sharing.
2017	[49]	Coverage capacity, outage probability	Indoor deployment and clustered resource allocation. Spectrum sharing.
2016	[50]	Minimized system power consumption.	Holistic framework for green C-RAN under the constraint of limited front hauls capacity for VM. Performed hybrid clustering by controlling power metric.
2016	[44]	Achieved improved system utility and throughput for large scale networks.	Interference-separation clustering-based game-theoretic solution. Worked on spectrum.
2012	[47]	Improved SINR of MUE and FUE is achieved. Regional Average Channel State (RACS) metric is proposed to estimate the weight of interference	Hybrid clustering based on interference graph (HCIG) is projected to reduce interference. The optimal clustering problem is identified as a MAX-K cut problem, and a heuristic algorithm has been proposed.

**Table 3:** Simulation parameters.

Parameters	Values
Bandwidth	10 MHz
Transmission Power of MBS	46 dBm
Transmission Power of Pico BSs (HSBS)	30 dBm
Transmission Power of Femto BSs (LSBS)	15 dBm
Channel Gain (MBS)	14 dBi
Channel Gain (SBS)	7 dBi
Indoor/Outdoor Path loss Coefficient	2
Radius of MBS (Macro BS)	500 m
Radius of HSBS (Pico BS)	25 m
Radius of LSBS (Femto BS)	10 m
Wall Penetration loss	6
No. of HSBS	100
No. of LSBS	1000

## Abbreviations

***Acronym***	***Description***
*BS*	*Base Station*
*SBS*	*Small Cell Base Station*
*MBS*	*Macro Cell Base Station*
*HSBS*	*High-Power Small Cell Base Station*
*LSBS*	*Low-Power Small Cell Base Station*
*UDN*	*Ultra-Dense Network*
*IMHC*	*Interference-Managed Hybrid Clustering*
*SPC*	*Small Cell Power Control*
*CH*	*Cluster Head*
*CM*	*Cluster Member*
*UE*	*User Equipment*
*SUE*	*Small Cell User Equipment*
*SGW*	*Small Cell Gateway*
*SIR*	*Signal To Interference Ratio*
*HetNet*	*Heterogeneous Network*
*PPP*	*Poisson Point Process*
*NOMA*	*Non Orthogonal Multiple Access*
*HetIot*	*Heterogeneous Internet-of-Things*
*RRM*	*Radio Resource Management*
*RAN*	*Radio Access Network*
